# Kratom-Associated Acute Right Ventricular Dysfunction: Diagnostic and Management Insights

**DOI:** 10.1016/j.jaccas.2025.104206

**Published:** 2025-07-23

**Authors:** Ibrahim Alameh, Maria V. Lebedev, John M. Clark, Mohammed Kamareddine, Daniele Ferrara, Olivia DeLorenzo, Bernita Vanessa Sidhu, Alyssa Heric

**Affiliations:** a Department of Internal Medicine, Lankenau Medical Center, Wynnewood, Pennsylvania, USA; b Philadelphia College of Osteopathic Medicine (PCOM), Philadelphia, Pennsylvania, USA; c Department of Cardiology, Lankenau Medical Center, Wynnewood, Pennsylvania, USA

**Keywords:** acute heart failure, cardiomypathy, right ventricle, systolic heart failure

## Abstract

**Background:**

Kratom (*Mitragyna speciosa*) is a psychoactive herbal supplement with stimulant and opioid-like properties, with emerging reports linking it to cardiovascular toxicity. However, its association with right ventricular (RV) function remains unclear.

**Case Summary:**

We report the case of a 52-year-old man who presented in shock with hypoxia and multiorgan dysfunction. Echocardiography revealed severe RV dilation with McConnell’s sign, whereas chest computed tomography angiography ruled out pulmonary embolism. Right heart catheterization confirmed elevated right-sided pressures and high cardiac output consistent with high-output RV failure.

**Discussion:**

Despite intermittent Kratom use, other etiologies, including thyrotoxicosis, arteriovenous fistula, liver disease, thiamine deficiency, and infection, were systematically excluded. With supportive management, including inotropic therapy, the patient’s RV function recovered.

**Take-Home Messages:**

This case emphasizes the importance of recognizing Kratom as a potential factor in unexplained acute RV dysfunction. It highlights the value of prompt echocardiographic and hemodynamic assessment in guiding effective management.

## Graphical Abstract

**Figure:**
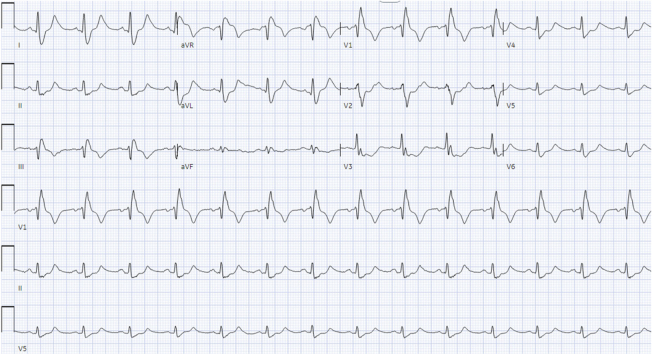

## History of Presentation

A 52-year-old man presented to the emergency department with acute onset shortness of breath and confusion. On arrival, he was hypoxic (80% on room air) and hypotensive (72/41 mm Hg). Physical examination revealed elevated jugular venous pressure, warm extremities, and no peripheral edema. His hypoxia improved with noninvasive ventilation, but his hypotension persisted despite intravenous fluids, requiring norepinephrine for hemodynamic support.

## Past Medical History

His medical history included anxiety and nonalcoholic fatty liver disease with normal baseline liver enzymes. He endorsed self-medicating for anxiety with intermittent Kratom and Delta-8-tetrahydrocannabinol use, with his last reported use being weeks before presentation. He denied prior cardiovascular or pulmonary disease or family history of cardiac disease.

## Differential Diagnosis

His acute presentation with hypoxia, shock, and confusion raised suspicion for massive pulmonary embolism, acute myocardial infarction, acute myocarditis, stress-induced cardiomyopathy, and toxin-induced cardiomyopathy, particularly from substances such as Kratom in his case. Other considerations include sepsis and high-output heart failure.

## Investigations

**Figure 1:**
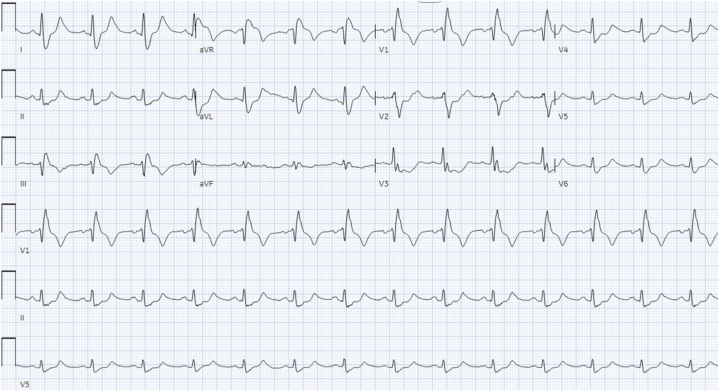
Electrocardiogram With Acute Right Heart Strain Pattern: McGinn-White Sign, Also Known as the “S1Q3T3” Pattern A large S-wave in lead I, a Q-wave in lead III, and an inverted T-wave in lead III.

**Figure 2:**
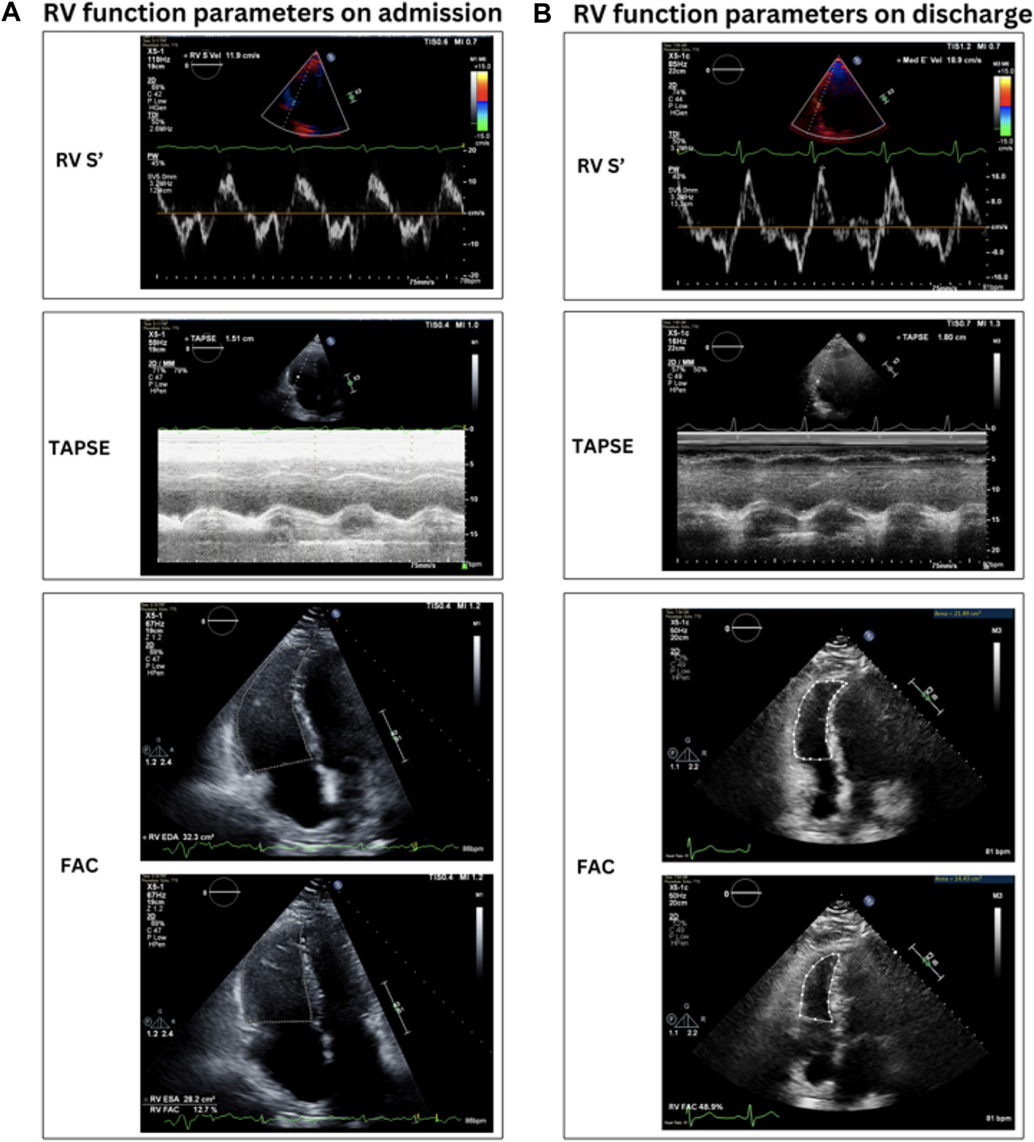
Transthoracic Echocardiogram Showing Right Ventricular Features Before and After Supportive Treatment (A) RV function parameters on admission: RV S′ velocity 11.9 cm/s, TAPSE 1.51, and FAC 12.7%. (B) RV function parameters before discharge: RV S′ velocity 18.9 cm/s, TAPSE 1.8, and FAC 48.9%. FAC = fractional area change; RV = right ventricle; RV S′ = peak lateral tricuspid annular systolic velocity; TAPSE = tricuspid annular plane systolic excursion.

Initial laboratory evaluation was significant for acute kidney injury (creatinine level of 1.9 mg/dL from baseline 0.9-1.0 mg/dL), hyperkalemia (potassium level of 5.4 mEq/L), acute liver injury (aspartate aminotransferase [AST] level of 758 U/L, alanine aminotransferase [ALT] level of 673 U/L), and mixed acidosis (lactic acid level of 4.0 mmol/L) (Table 1). His urine drug screening was negative for opioids. An electrocardiogram revealed sinus tachycardia, a right bundle branch block, and findings concerning right ventricular strain (Figure 1). An urgent transthoracic echocardiography (TTE) demonstrated severe right ventricular (RV) dilation, reduced systolic function, McConnell’s sign, and preserved left ventricular function with an ejection fraction >75% (Figure 2A, Video 1). Computed tomography angiography of the chest was negative for pulmonary embolism. Troponin levels rose markedly overnight (152 to 9,624 ng/L), with worsening kidney and liver function (creatinine level to 2.8 mg/dL, AST level > 5,000 U/L, and ALT level > 2,500 U/L). Additional investigations, including thyroid function tests and Doppler ultrasound of extremities, were within normal limits.

**Table 1:** Initial Laboratory Values Upon Patient Admission

	Labs on Admission	Reference Ranges
WBC (neutrophils %)	9,770 (87%)	3,800-10,500
pH (venous blood gas)	7.19	7.35-7.45
Lactic acid (mmol/L)	4.0	0.4-2.0
Sodium (mEq/L)	137	136-145
Potassium (mEq/L)	5.4	3.5-5.1
Chloride (mEq/L)	97	98-107
Carbon dioxide (mEq/L)	30	21-31
Anion gap (mEq/L)	10	3-15
Glucose (mg/dL)	83	70-99
Blood urea nitrogen (mg/dL)	13	7-25
Creatinine (mg/dL)	1.9	0.7-1.3
Albumin (g/dL)	3.9	3.5-5.7
AST (IU/L)	758	13-39
ALT (IU/L)	673	7-52
Alkaline phosphatase (IU/L)	48	34-125
Bilirubin total (mg/dL)	1.4	0.3-1.2
Calcium (mg/dL)	8.6	8.6-10.3
Magnesium (mg/dL)	2.1	1.8-2.5
High-sensitivity troponin (pg/mL)	152.0	<15
Thyroid-stimulating hormone (mIU/L)	1.91	0.34-5.60

ALT = alanine aminotransferase; AST = aspartate aminotransferase; WBC = white blood cell count.

## Management

Because of persistent hemodynamic instability, severe RV dysfunction, and multiorgan failure, he was started on dobutamine and transferred to the cardiac care unit. Given the unclear etiology of acute right ventricular failure, the patient underwent urgent heart catheterization and evaluation for possible RV assist device placement. Coronary angiography showed minimal luminal irregularities on day 2 of the hospital stay. Right heart catheterization revealed elevated right-sided pressures (right atrial pressure of 24 mm Hg, RV pressure of 56/15 mm Hg [mean 27 mm Hg], pulmonary artery pressure of 56/31 mm Hg [mean 40 mm Hg], and pulmonary capillary wedge pressure of 27 mm Hg) and a cardiac output and cardiac index of 13.4 L/min and 5.0 L/min/m^2^, respectively. A serum thiamine level was sent, and intravenous supplementation was initiated during hospitalization; subsequent results demonstrated an elevated concentration of 77 nmol/L (reference range: 8-30 nmol/L), effectively excluding thiamine deficiency as a contributing factor. On day 3, TTE showed a decreased RV size and improved systolic function, though not fully normalized (Figure 2B, Video 2), allowing discontinuation of vasopressors. Over the next several days, diuresis was held because of concerns of hemodynamically mediated acute kidney injury, with blood pressures intermittently dropping to 70-79/40-49 mm Hg and oxygen saturation to 89%, requiring supplemental oxygen via a nasal cannula. Despite holding diuresis, the creatinine level continued to rise, peaking at 4.3 mg/dL. Midodrine was initiated for blood pressure support and titrated to 10 mg twice daily. With improving blood pressure stability, the creatinine level declined to 3.4 mg/dL 2 days later.

## Discussion

Kratom (*Mitragyna speciosa*) is a tropical tree native to Southeast Asia and Africa whose leaves have been used for centuries as a traditional remedy. It has μ-opioid and adrenergic activity.^1^ It has recently been used for its effects as an aphrodisiac, opioid substitute, anxiolytic, antidepressant, stimulant, and sedative. Its rising use in the United States has been associated with hepatotoxicity, neurologic effects, and, more recently, cardiovascular complications, including arrhythmia and cardiomyopathy.^2^

In recent years, Kratom has gained popularity in Western countries, with an estimated 1.7 million Americans 12 years and older using Kratom in 2021, according to the Substance Abuse and Mental Health Services Administration’s National Survey on Drug Use and Health,^3^ often using it for chronic pain relief or to self-manage opioid withdrawal. Despite its perception as a “natural” and safe alternative, the Food and Drug Administration has not approved Kratom for any medical use, and numerous cases of severe toxicity have been reported. Kratom’s pharmacologically active alkaloids (primarily mitragynine and 7-hydroxy mitragynine) act on opioid receptors and have stimulant properties. It has complex cardiovascular effects that vary with dose. At low to moderate doses, it can have mild stimulant effects, whereas higher doses produce opioid-like sedation, which might blunt heart rate; however, in practice, tachycardia and hypertension are by far the most observed effects, even in routine Kratom use.^4^ These effects are usually transient and mild, but they signal Kratom’s impact on the autonomic nervous system. Less commonly, Kratom has been associated with other cardiovascular signs such as arrhythmias or even hypotension in some cases.

**Figure 3:**
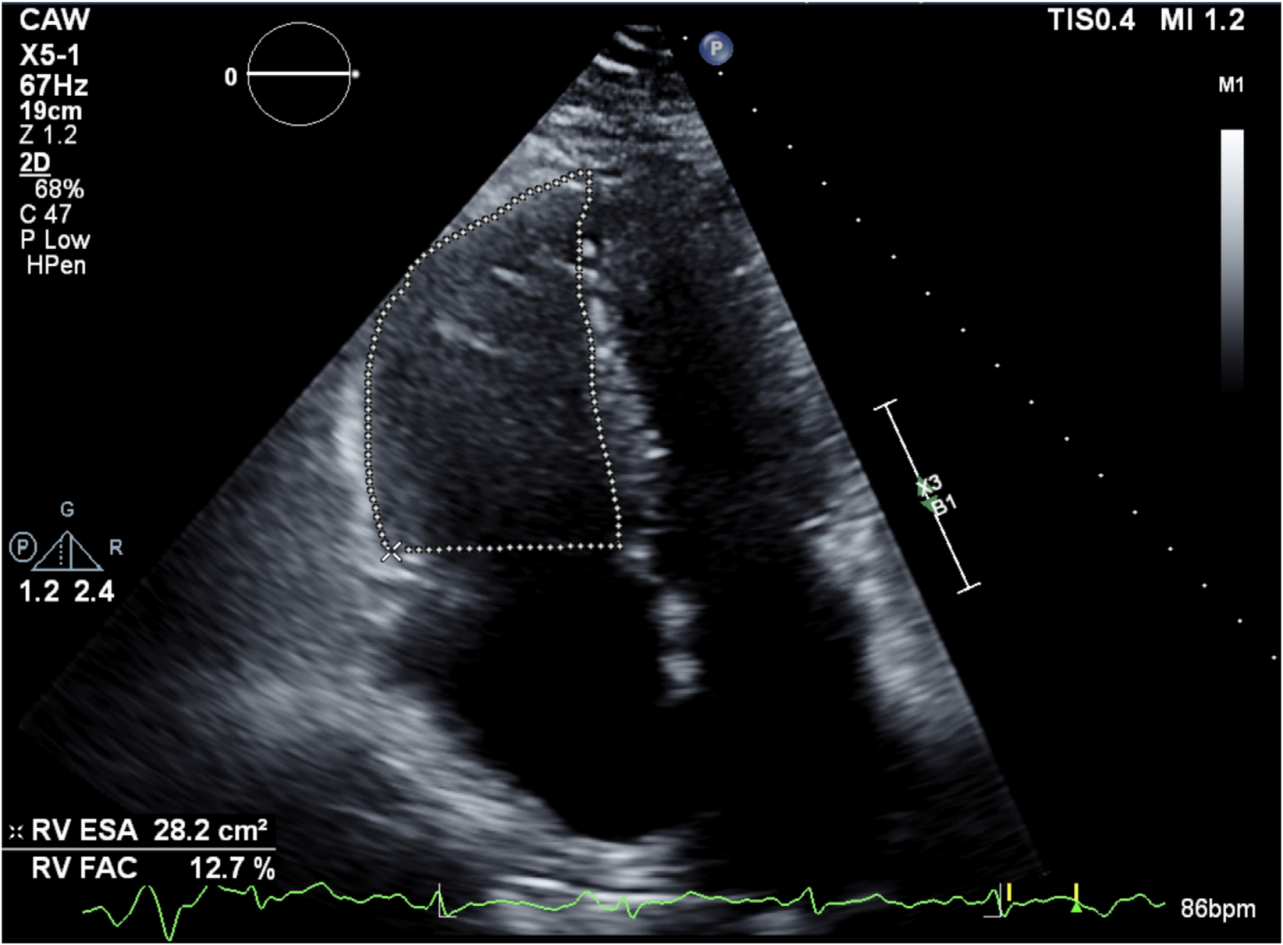
Right Ventricular Fractional Area Change on Admission Measured at 12.7% ESA = end-systolic area; FAC = fractional area change; RV = right ventricular.

In addition to these hemodynamic effects, Kratom use has been linked to specific electrocardiogram changes. Notably, Kratom’s alkaloids can prolong cardiac repolarization—prolonged QT intervals have been observed in both experimental and clinical settings. In vitro studies showed that mitragynine can significantly prolong the cardiac action potential duration, indicating a risk for QT interval prolongation and torsades de pointes (a dangerous ventricular arrhythmia).^4^ Clinically, a case series of regular Kratom users in Malaysia found that those with higher blood levels of mitragynine tended to have longer (borderline) QTc intervals on electrocardiogram.^5^ Significantly, none of those individuals developed torsades or fatal arrhythmias in that study. Their TTEs were largely within normal limits. In contrast, our case demonstrated a dilated right ventricle (RV > left ventricle in size), with a reduced tricuspid annular plane systolic excursion (<16 mm), consistent with impaired RV systolic function seen in Figures 2 and 3. Nevertheless, the dose-dependent QT prolongation effect suggests that heavy Kratom consumption could predispose susceptible users to arrhythmias.

The rise in Kratom usage, particularly in North America, has introduced a new set of health risks. Kratom remains legal in many areas and easily obtainable (online or in convenience stores), which fosters a misconception of safety among users.^4^ As highlighted above, Kratom can cause serious harm, including organ failure and cardiac emergencies. The past decade has seen Kratom use surge because of its availability and the opioid epidemic (people seeking a “safer” opioid alternative). This trend is concerning to health care professionals and toxicologists, who view Kratom as a potential public health threat, given the severity of some reported cases. Greater public education is needed to dispel the myth that “natural” equals safe.

For most casual Kratom users, the immediate cardiovascular effects might be limited to tachycardia and hypertension,^4^ whereas, as seen in our patient, serious complications of RV dysfunction can occur. However, the risk spectrum is broad—from mild palpitations to life-threatening arrhythmias or cardiomyopathy.^6^, ^7^, ^8^, ^9^ It appears that dose and purity matter: high doses or adulterated products markedly increase the danger. Even in the absence of codrugs, a heavy binge of Kratom can stress the cardiovascular system to the point of collapse. Thus, its cardiovascular toxicity, while not ubiquitous, can be severe and unpredictable.

In addition, Kratom’s stimulant-like action can create a hyperadrenergic state, which might precipitate something akin to stress cardiomyopathy in extreme cases. There is also interest in whether Kratom might directly damage cardiac myocytes or vasculature through oxidative stress or other pathways. Currently, no strong evidence of direct myocardial necrosis from Kratom itself has been established (beyond some autopsy correlations).

In evaluating our patient’s high-output heart failure, common etiologies were systematically evaluated and excluded. The results of thyroid function tests were within normal limits, excluding thyrotoxicosis as a potential cause. Doppler ultrasound demonstrated no significant arteriovenous fistulas or shunts that could contribute to high-output physiology. Liver imaging and biochemical tests excluded advanced hepatic dysfunction or cirrhosis. In addition, anemia, systemic infection, and thiamine deficiency were clinically and laboratory-wise absent. Thus, the comprehensive exclusion of alternative high-output states further supports a toxic mechanism, possibly associated with prior Kratom exposure in this patient.

## Follow-Up

On day 6, the patient was transferred from the intensive care unit off diuretics and midodrine with stable blood pressure. He was discharged with a creatinine level of 1.3 mg/dL, an AST level of 64 U/L, an ALT level of 466 U/L, and a complete resolution of symptoms. He was ultimately discharged home without any medications, with instructions to abstain from Kratom use.

## Conclusions

We present a rare case of acute right ventricular failure associated with heavy Kratom use, expanding the clinical spectrum of Kratom’s cardiovascular toxicity and highlighting its potential to cause acute RV failure in the absence of coronary or pulmonary thromboembolic disease. With increasing Kratom use, clinicians should maintain a high index of suspicion for its cardiovascular effects, especially in younger patients with unexplained RV dysfunction. Ongoing surveillance and research into Kratom’s safety profile is warranted, given its growing use. In summary, Kratom use can precipitate severe cardiovascular events in certain individuals, and our case particularly highlights the possibility of acute RV failure—an association that clinicians and consumers alike should be aware of.

**Take-Home Messages**

• Kratom use may precipitate isolated, acute right ventricular failure without pulmonary embolism or coronary disease.
• Prompt echocardiography and invasive hemodynamics are essential to guide diagnosis and management in such patients.

## Funding Support and Author Disclosures

The authors have reported that they have no relationships relevant to the contents of this paper to disclose.

## Appendix

Video 1 Echocardiographic 4-Chamber View Showing Severely Dilated Right Ventricular With Severe Systolic Dysfunction and Apical Hypercontractility (Positive McConnell’s Sign) Fractional area change was measured at 12.7%.

Video 2 Echocardiographic 4-Chamber View Showing Right Ventricular Recovery With Improved Systolic Function, Now Mildly Decreased Fractional area change was measured at 48.9%.
